# A New Chapter for *Nanophotonics*

**DOI:** 10.1002/nap2.70004

**Published:** 2026-01-13

**Authors:** Nadezda Panarina

**Affiliations:** ^1^ Wiley‐VCH GmbH Berlin Germany

*Nanophotonics* has always been more than just a publication—it is a platform for collaboration, innovation and the exchange of ideas that drive the photonics field forward. As the new Editor‐in‐Chief of *Nanophotonics*, I am honoured to continue the journal's tradition of excellence and its strong connection to the scientific community.

Today, we are beginning an exciting new chapter in the journal's development as we publish the first *Nanophotonics* issue at Wiley. Although this transition brings a new editorial structure (in‐house instead of an external editorial team), our commitment to quality and scientific rigour remains unchanged. I would like to express my gratitude to the previous editorial team, especially Dennis Couwenberg and Tara Dorrian, whose dedication has laid a solid foundation for the journal's success. We are also privileged to have the continued support of the former Editor‐in‐Chief Stefan Maier, as well as managing editors Tie Jun Cui, Antonio I. Fernández‐Domínguez, Dangyuan Lei, Yongmin Liu, Matthew Sheldon and Giulia Tagliabue, who will now be contributing as members of the **Executive Editorial Board**. Their expertise and guidance will ensure continuity and provide stability as we embrace new opportunities and move forward.

There are indeed many exciting opportunities for the journal as part of the Wiley family. Alongside leading Wiley photonics titles such as *Laser & Photonics Reviews* and *Advanced Optical Materials*, *Nanophotonics* will continue to deliver high‐quality content, ensuring that the best photonics research is easily accessible to the global scientific community. We welcome collaborations that strengthen our mission, and we invite you to engage with us as we shape the future of nanophotonics research.

Wiley itself is evolving, introducing improvements that benefit both authors and readers. One of the most significant changes is the launch of the new Research Exchange submission system, which is designed to make the submission process more intuitive and author‐friendly. This innovation reflects our shared goal of making publishing as seamless and rewarding as possible.

I am also delighted to announce that the ***Nanophotonics* Early Career** **Awards** will continue. These awards celebrate emerging talent and recognise the contributions of young researchers. The call for applications is open until mid‐March 2026, and I encourage you to put forward outstanding candidates who represent the next generation of leaders in the field.

Thank you for your trust and support. I look forward to working with all of you to ensure that *Nanophotonics* remains a vibrant and influential voice in the photonics community.

## Author Contributions

**Nadezda Panarina:** writing – original draft.

## Funding

The author has nothing to report.

## Data Availability

The author has nothing to report.

